# The relationship among university students’ recreational flow experience, psychological resilience, social connectedness, and life satisfaction

**DOI:** 10.3389/fpsyg.2026.1774124

**Published:** 2026-04-24

**Authors:** Sevim Güllü, Yunus Şahinler, Ayça Genç, Elif Bozyiğit, Selin Biçer Baikoğlu, Neşe Genç, Orkun Akkoç

**Affiliations:** 1 Faculty of Sport Science, Istanbul University-Cerrahpasa, Istanbul, Türkiye; 2 Faculty of Sport Science, Gelisim University, Istanbul, Türkiye; 3 Faculty of Sport Science, Bartın University, Bartın, Türkiye; 4 Faculty of Sport Science, Pamukkale University, Denizli, Türkiye; 5 Ministry of National Education, Adana, Türkiye

**Keywords:** flow experience, life satisfaction, positive psychology, psychological resilience, recreation, social connectedness, subjective well-being

## Abstract

**Introduction:**

This study aimed to examine the relationships among recreational flow experience, social connectedness, psychological resilience, and life satisfaction in university students.

**Methods:**

The sample consisted of 810 (416 female, 394 male) university students from various universities in Istanbul who voluntarily participated in the study. Data were collected using four standardized measurement instruments: the Recreational Flow Experience Scale, the Social Connectedness Scale, the Brief Resilience Scale, and the Satisfaction with Life Scale. Statistical analyses were conducted using SPSS and Hayes’ PROCESS Macro (Model 4) to test both direct and indirect association among the variables.

**Results:**

Recreational flow significantly is positively associated with social connectedness and psychological resilience. Social connectedness and psychological resilience each is positively associated with life satisfaction. Furthermore, the relationship between recreational flow and life satisfaction was accounted for by a parallel mediation structure, in which social connectedness and psychological resilience operated as complementary pathways.

**Discussion:**

These results indicate that both social and psychological processes contribute to explaining how recreational flow relates to life satisfaction. The study highlights the distinct roles of social connectedness and psychological resilience in strengthening the positive outcomes of recreational flow and provides empirical evidence on the interconnections among these four constructs in the university student population.

## 1 Introduction

University students constitute a population particularly vulnerable to fluctuations in well-being and life satisfaction due to academic demands, social transitions, and reduced access to stable support systems. During this developmental period, students are required to regulate stress, establish new social bonds, and maintain psychological balance while adapting to increased autonomy and responsibility. In line with these vulnerabilities, large-scale international evidence shows that mental health problems are common in university populations: in the WHO World Mental Health International College Student Project, 31% of first-year students screened positive for at least one 12-month DSM-IV mental disorder across multiple countries (Auerbach et al., 2018). Similarly, recent national evidence indicates that mental health difficulties are widespread among university students, with approximately one-third reporting moderate to severe anxiety symptoms and around four in ten reporting moderate to severe depressive symptoms in large-scale assessments of student well-being [Healthy Minds Study (HMS), 2025]. These patterns underscore the importance of examining experiential and psychosocial factors associated with sustainable well-being and life satisfaction among university students.

In the present study, “well-being” is approached through the lens of positive psychology and subjective well-being (SWB). Life satisfaction represents the cognitive evaluation component of SWB and reflects individuals’ overall judgment of their quality of life (Diener, 1984). Within positive psychology, flourishing is conceptualized as a multidimensional state of optimal functioning encompassing positive emotions, engagement, relationships, meaning, and accomplishment (Seligman, 2011). Importantly, flourishing is not treated here as a single synonymous outcome with “what makes life worth living”; rather, it is used as an overarching framework that emphasizes a constellation of psychological capacities and experiences that make life more fulfilling (Agarwal, 2020; Chou et al., 2013; Franco, 2020; Liu, 2023). In this conceptual framing, life satisfaction is positioned as a core cognitive indicator of well-being that can be influenced by experiential and psychosocial resources.

Flow refers to a state of optimal experience characterized by deep absorption, focused attention, intrinsic enjoyment, and a balance between perceived challenges and individual skills (Csikszentmihalyi, 1974). Recreational flow experience is increasingly considered a relevant contributor to student well-being, as such experiences may occur during intrinsically motivated leisure activities (e.g., sports, arts, or nature-based activities). Experiencing flow during recreational engagement may provide psychological restoration by replenishing mental energy and helping students manage daily pressures (Liao et al., 2023). Empirical evidence also suggests that frequent engagement in flow experiences is associated with higher life satisfaction and a stronger sense of meaning among students (Ding et al., 2023). Recreational flow may be associated with life satisfaction alongside interpersonal and intrapersonal psychosocial resources such as social connectedness and psychological resilience (Mao et al., 2024; Özsoy et al., 2025).

Among these psychosocial resources, social connectedness is central to university students’ mental health and well-being, reflecting feelings of belonging, trust, and meaningful interaction with peers (Bianchi and Martini, 2023). Social connectedness goes beyond superficial contact and captures perceived inclusion and support within a social network (Xie et al., 2022). As students often experience reduced direct family support during the university years, social connectedness may function as a compensatory emotional resource (Jusri and Lechner, 2025). Recreational activities frequently occur in socially interactive environments in which individuals participate alongside others, share experiences, and engage in informal peer interaction. Such contexts may naturally create opportunities for social connection and a sense of belonging among participants. In the present study, recreational flow is conceptualized as an individual experiential state that may occur during recreational engagement, rather than as a collective or socially generated psychological process. Therefore, the present research does not assume that the psychological state of flow itself produces social connectedness. Instead, the study examines whether students who report higher levels of recreational flow also report stronger perceptions of social connectedness within recreational contexts. In this respect, the observed association may reflect the socially interactive nature of recreational participation rather than a direct psychological mechanism linking flow to social belonging.

Psychological resilience—defined as the capacity to adapt positively to stress and adversity—is another mechanism through which recreational flow may support sustainable well-being (Mao et al., 2024). Resilience reflects emotional stability, recovery after setbacks, and growth through challenges (Schwarz, 2018). For university students facing academic, social, and financial stressors, resilience is a key predictor of mental health and life satisfaction (Gómez Molinero et al., 2018), and importantly, it can be strengthened through positive experiences (Luthar et al., 2014). Recreational flow provides structured challenges that may enhance self-efficacy and support adaptive regulation, thereby strengthening resilience (Decloe et al., 2009). Social resources can further support coping by reducing isolation and fostering adaptive responses (Hou et al., 2024). Hence, resilience can be conceptualized as both an outcome of flow-oriented recreation and a mediator linking experiential engagement to higher life satisfaction (Deng et al., 2023).

Despite growing interest in recreational experiences and student well-being, limited research has examined how recreational flow relates to life satisfaction through both interpersonal and intrapersonal psychosocial resources among university students. Building on positive psychology and subjective well-being (SWB), the present study develops and tests a parallel mediation model in which recreational flow is hypothesized to be associated with life satisfaction, both directly and indirectly through social connectedness and psychological resilience. By integrating an experiential construct (recreational flow) with interpersonal (connectedness) and intrapersonal (resilience) resources, the model aims to contribute to the growing evidence on leisure-based well-being mechanisms in university student populations.

## 2 Literature review and hypothesis development

Previous research has demonstrated that participation in campus recreation programs is associated with a variety of positive outcomes for university students. Studies indicate that recreational sport participation on campus contributes to students’ sense of belonging, social integration, and emotional connection to the university environment (Miller, 2011; Phipps et al., 2015). In addition, campus recreation engagement has been linked to higher levels of student engagement and academic adjustment, particularly during the transition to university life (Mayers et al., 2017). From an institutional perspective, recreational facilities and programs also play an important role in student recruitment and retention by enhancing students’ overall campus experience and well-being (Kampf et al., 2018). Together, these findings suggest that campus recreation environments provide meaningful contexts for social interaction, personal development, and university integration among students. Within such recreational contexts, students may also encounter experiential states such as flow, which have been associated with various indicators of well-being in previous research.

### 2.1 Recreational flow and social connectedness

Flow refers to an optimal psychological state characterized by deep concentration, intrinsic motivation, and a loss of time awareness during an activity (Csikszentmihalyi, 1990; Beard, 2015). Flow experiences can occur in both work and leisure contexts, although they are often more frequently reported in leisure activities (Csikszentmihalyi and LeFevre, 1989). Importantly, in the present study, recreational flow is conceptualized as an individual experiential state, rather than as a collective or group-level psychological phenomenon. When experienced in socially interactive recreational settings, flow may occur alongside opportunities for interpersonal contact and shared activities among participants. These experiential features are conceptually aligned with social connectedness, which reflects perceived belonging, inclusion, and supportive peer relationships (Xie et al., 2022), particularly during the university years when peer networks constitute a primary source of social support (Jusri and Lechner, 2025). Within recreational participation more broadly, students may experience varying levels of experiential engagement during leisure activities, including flow-like absorption. Importantly, the present study does not assume that the psychological state of flow itself directly produces social connectedness. Rather, the study examines whether students who report higher levels of recreational flow during their leisure activities also report higher levels of perceived social connectedness. In this sense, the association between these variables may reflect the socially interactive nature of recreational participation more broadly rather than a direct psychological mechanism linking flow to social belonging. Empirical research has also reported associations between engaging leisure experiences and indicators of social well-being. For example, leisure-related flow has been linked to lower loneliness among international university students (Chang et al., 2024). Accordingly, the present study examines whether recreational flow is statistically associated with perceived social connectedness among university students.

*H1*: Recreational flow is positively associated with perceived social connectedness.

### 2.2 Recreational flow and psychological resilience

Flow experiences are often elicited by contexts that provide optimal challenges and opportunities for mastery (Csikszentmihalyi, 1974). Such structured challenges may strengthen adaptive regulation and perceived competence, which are core elements in resilience processes (Decloe et al., 2009; Schwarz, 2018). In university settings, resilience is particularly consequential for sustaining well-being under academic and social demands (Gómez Molinero et al., 2018; Luthar et al., 2014). Recent empirical findings also support a positive association between recreational flow and psychological resilience, indicating that students with higher flow in recreational activities tend to report stronger resilience (Özsoy et al., 2025). Thus, recreational flow is expected to be positively associated with psychological resilience.

*H2*: Recreational flow is positively associated with psychological resilience.

### 2.3 Social connectedness and life satisfaction

Social connectedness is a robust correlate of students’ well-being, as it reflects perceived belonging, support, and meaningful peer relationships (Bianchi and Martini, 2023). Given that the university years may involve diminished direct family support, connectedness can function as a compensatory emotional resource that supports psychological adjustment (Jusri and Lechner, 2025). Empirical work demonstrates that social connectedness explains life satisfaction beyond demographic factors and that increases in connectedness correspond to increases in life satisfaction (Avcı, 2023; Blau et al., 2016). Therefore, social connectedness is expected to be positively associated with life satisfaction.

*H3*: Social connectedness is positively associated with life satisfaction.

### 2.4 Psychological resilience and life satisfaction

Resilience supports positive adaptation under stress and is strongly linked to favorable evaluations of life circumstances and personal functioning (Schwarz, 2018). Among university students, resilience is consistently associated with higher life satisfaction, suggesting that students who can recover and adapt effectively tend to evaluate their lives more positively (Al-Abyadh and Azim, 2020). Because resilience can be strengthened through positive experiences (Luthar et al., 2014) and is supported by social resources (Hou et al., 2024), it is positioned as a central intrapersonal pathway to life satisfaction in the present model.

*H4*: Psychological resilience is positively associated with life satisfaction.

### 2.5 Parallel mediation of social connectedness and psychological resilience

Recreational flow can be expected to influence life satisfaction both directly and indirectly. Flow-rich leisure experiences are positively associated with life satisfaction in student samples, indicating that optimal experiential engagement can translate into more favorable global life evaluations (Hou and Jiang, 2023; Ding et al., 2023). At the same time, flow can foster interpersonal resources (connectedness) and intrapersonal resources (resilience), both of which independently contribute to life satisfaction (Blau et al., 2016; Al-Abyadh and Azim, 2020). Because social connectedness and resilience represent distinct but complementary mechanisms—belonging-related support and adaptive coping capacity—the present study tests them simultaneously as parallel mediators linking recreational flow to life satisfaction.

*H5a*: Social connectedness mediates the relationship between recreational flow and life satisfaction.

*H5b*: Psychological resilience mediates the relationship between recreational flow and life satisfaction.

## 3 Material and method

### 3.1 Study design

This study employed a cross-sectional correlational research design. As defined by Karasar (2011), general survey models aim to derive generalized conclusions about a population comprising numerous elements by examining either the entire population or a sample drawn from it.

Data were collected during scheduled class sessions across multiple faculties located on the European side of Istanbul between September and October 2025. Students completed the online questionnaire individually using a survey link or QR code provided by the researcher. All responses were obtained in classroom environments under similar administration conditions, ensuring procedural consistency across participants.

### 3.2 Participants and sampling

A total of 810 university students (416 females, 394 males) participated in the study, recruited through non-probability convenience sampling. Data were gathered from students studying in various faculties of different universities located on the European side of Istanbul. Participants were recruited from multiple faculties across different universities located on the European side of Istanbul. Although participants were enrolled in different institutions, data collection took place within classroom environments at their respective faculties during scheduled class sessions. Thus, the institutional origin of participants and the physical setting of data collection were distinct but related aspects of the sampling process.

The inclusion criteria were as follows: (i) being 18 years of age or older, (ii) being currently enrolled as a university student, (iii) voluntary participation and provision of electronic informed consent, and (iv) completion of all required questionnaire sections. The exclusion criteria included: (i) incomplete or invalid responses, and (ii) participants who did not meet the inclusion requirements. A total of 810 responses were collected, and all questionnaires were complete and met the inclusion criteria. Therefore, no cases were excluded during the data screening process, and all responses were retained for statistical analyses.

Data were screened for missing values, outliers, and distributional assumptions prior to analysis. The proportion of missing data was minimal and below recommended thresholds. Outliers were evaluated using standardized z-scores and multivariate distance indicators, and no influential cases were detected. Normality was assessed using skewness and kurtosis statistics, and all variables fell within acceptable ranges. Participants were recruited using a convenience sampling strategy. Data were collected during scheduled class sessions across multiple faculties, and participation was voluntary. All data were collected using a single administration mode via an online questionnaire. The term “in-class administration” refers to the context in which students completed the online survey during scheduled class sessions using a survey link or QR code; no paper-based data collection was conducted.

### 3.3 Data collection tools

Participants first provided sociodemographic information and answered structured questions. They were asked questions regarding gender, average daily leisure time, preferred companions during recreational activities (e.g., individual, with family, with friends), and types of recreational physical activities they most frequently engaged in.

#### 3.3.1 Measurement instruments and psychometric properties

Four standardized measurement tools were used in this study: Each scale was administered using standardized instructions, and higher scores indicated higher levels of the measured construct. Example items are provided to illustrate the measurement context.

- Recreational Flow Experience Scale (RFES): Developed by Ayhan et al. (2020), the RFES consists of 9 items rated on a 7-point Likert scale (1 = strongly disagree, 7 = strongly agree). The scale assesses self-reported recreational flow characteristics such as absorption, focused attention, enjoyment, and time transformation experienced during recreational activities. Example items include: “I feel completely absorbed in the activity I am doing,” “I give my full attention to the activity,” and “I lose track of time during the activity.” Following the scoring procedure proposed by the scale developers, the RFES score is calculated as the mean of the item responses, with higher mean scores indicating higher levels of recreational flow experience (Ayhan et al., 2020). The original scale development study reported satisfactory construct validity and reliability. In the present study, internal consistency reliability was high (Cronbach’s *α* = 0.923). RFES scores were treated as continuous indicators of self-reported flow experience in line with the scoring approach proposed by the scale developers. The RFES has also been applied in subsequent studies examining recreational participation and related psychological variables (e.g., nature relatedness, leisure attitude, intrinsic motivation, and boredom), providing further evidence of its empirical use in recreational research (Akçakese et al., 2024; Ayhan and Eskiler, 2024; Bedir, 2023; Emir and Yıldız, 2023).
- Brief Resilience Scale (BRS): Developed by Smith et al. (2008) and validated for the Turkish context by Doğan (2015), this 6-item, 5-point Likert-type scale (1 = strongly disagree, 5 = strongly agree) measures the ability to recover from stress and adversity, reflecting psychological resilience as a dynamic adaptive capacity. Example item: “I tend to bounce back quickly after difficult times.” Some items are reverse scored. Higher total scores indicate greater psychological resilience. Previous studies support its construct validity and reliability. Internal consistency in the present sample was acceptable (Cronbach’s *α* = 0.750). The scale has been validated in diverse adult and university student samples.
- Life Satisfaction Scale (LSS): Developed by Diener et al. (1985) and adapted into Turkish by Köker (1991), this scale comprises 5 items rated on a 7-point Likert scale (1 = not at all appropriate, 7 = very appropriate). It measures individuals’ overall cognitive evaluations of life satisfaction as one of the key indicators of subjective well-being. Example item: “In most ways my life is close to ideal.” Higher scores reflect higher life satisfaction. Prior research supports its factorial and construct validity. Internal consistency in the present sample was good (Cronbach’s *α* = 0.853). The scale has been extensively validated across student and adult populations worldwide.
- Social Connectedness Scale (SCS): Originally developed by Lee and Robbins (1995) and adapted into Turkish by Duru (2007), this scale consists of 8 negatively worded items (e.g., “I feel disconnected from the people around me,” “I do not feel a sense of togetherness with my friends”) rated on a 6-point Likert scale (1 = strongly agree, 6 = strongly disagree).
  To ensure consistency across measures, the response anchors were reversed during administration (“1 = strongly disagree” to “6 = strongly agree”) so that all scales followed the same positive response direction. Before data analysis, all SCS items were reverse-coded to align with the original scoring procedure, in which higher total scores indicate stronger social connectedness. This adjustment was implemented to minimize respondent confusion and enhance measurement reliability. Example item: “I feel disconnected from the people around me.” Items are reverse worded and reverse coded prior to analysis. Higher scores indicate stronger social connectedness. Previous studies support its construct validity. Internal consistency in the present sample was high (Cronbach’s *α* = 0.883).

The scales have been reported to demonstrate acceptable levels of construct validity and reliability in previous adaptation and validity studies. The internal consistency coefficients calculated for the current sample are also at a sufficient level (see Table 1). These findings indicate that the measurement instruments are suitable for measuring the relevant constructs in university students. The scale has been widely validated in university student populations across different cultural contexts. The Social Connectedness Scale includes reverse-coded items. Prior to analysis, these items were recoded and total scores were computed according to the original scoring procedure. Due to the scoring direction, higher total scores represent stronger perceived social connectedness. The direction of regression coefficients involving SCS therefore reflects the numerical scoring structure rather than the conceptual direction of association. The scale has demonstrated strong psychometric properties in prior validation studies, including satisfactory internal consistency and construct validity in university student samples. In the present study, internal consistency was also high (Cronbach’s α = 0.883), indicating that the scale reliably measures perceived social connectedness in the current sample. Prior to analysis, the dataset was systematically screened for data quality and statistical assumptions. Missing data were examined at both item and case levels. The proportion of missing values was negligible (< 1%) and did not require imputation. All available responses were retained for analysis. Outliers were evaluated using standardized z-scores and multivariate distance indicators. Cases exceeding the conventional threshold of |z| > 3.29 were examined; however, no influential univariate or multivariate outliers were identified. Therefore, no cases were removed. Normality was assessed using skewness and kurtosis statistics for each study variable. All values fell within recommended ranges (skewness and kurtosis within ±3), indicating approximate univariate normality. In addition, mediation analysis was conducted using bootstrapping procedures, which do not require strict normality assumptions. These procedures confirm that the dataset met acceptable data quality and distributional criteria for regression-based mediation analysis.

**Table 1:** Descriptive statistics of variables, skewness and kurtosis values.

Variables	X	Skewness	Kurtosis	Cronbach Alpha
RFES	47,946	−0.710	2.067	0.923
SCS	18,029	0.949	0.378	0.883
BRS	19,482	−0.28	1.355	0.750
LSS	22,346	−0.236	−359	0.853

### 3.4 Data analysis

This study was conducted using a quantitative, descriptive (correlational) research design. The study examined the direct and indirect relations among recreational flow experience (RFES), self-connectedness (SCS), psychological resilience (BRS) and life satisfaction (LSS) variables in university students. The sample of the study consisted of 810 university students (416 females, 394 males) studying in the Faculties of Sport Sciences at various universities in Istanbul during the fall semester of 2025. The gender distribution was balanced (Female = 51.4%; Male = 48.6%). Participants were selected through the convenience sampling method. Data were analyzed using the SPSS 26.0 statistical software. Initially, the dataset was examined for missing or outlier values. Then, a normality test was conducted to evaluate the distributional characteristics of the variables. The obtained skewness and kurtosis values were found to be within the ±3 range, indicating that the data were normally distributed (Kline, 2011; George and Mallery, 2019). Frequency and percentage analyses were performed to describe the demographic characteristics of the participants. To determine the relations among the variables, the Hayes (2022) PROCESS macro Model 4 was used to test the parallel mediation roles of SCS and BRS in the relationship between RFES and LSS. The bootstrap resampling method (n = 5,000) was applied, and 95% confidence intervals (CIs) were calculated to assess the significance of indirect effects. Bootstrapping procedures implemented in Hayes’ PROCESS macro do not require the assumption of normality, as statistical inference is based on resampled confidence intervals. Bootstrap resampling (5,000 samples) was used to estimate confidence intervals for indirect effects. Although the data were normally distributed and the sample size was large, indirect effects in mediation models often do not follow a normal sampling distribution. Therefore, bootstrap methods were employed to obtain more accurate confidence interval estimates without relying on normality assumptions. This approach provides more robust inference compared to traditional methods such as single-sample regression or the Sobel test and is widely recommended for mediation analysis. To assess potential common method variance, a full collinearity test was conducted following Kock (2015). Variance inflation factor (VIF) values were calculated for all study constructs. The study employed regression-based mediation analysis using Hayes’ PROCESS macro rather than a structural equation modeling framework. As PROCESS estimates observed-variable models rather than latent measurement models, a separate confirmatory factor analysis of a measurement model was not required. Measurement validity was supported through the use of previously validated instruments and satisfactory internal consistency estimates in the current sample.

## 4 Results

An analysis of the daily leisure time distribution among 810 university students (51.4% were female and 48.6% were male) revealed that the majority of students have a moderate amount of daily leisure time, with the largest proportion engaging in 3–4 h of leisure activities per day. An examination of the types of physical activities practiced by participants revealed a diverse range of engagement patterns. The most common activity was ball sports such as football, basketball, and volleyball, practiced by 26.3% of the students. This was followed by running or walking, which accounted for 22.2% of the sample, and weight training activities (e.g., fitness, bodybuilding) with 18.3% participation. Smaller proportions of students engaged in racket sports such as tennis, table tennis, or badminton (5.4%), combat sports including taekwondo, wushu, and karate (4.8%), and water sports such as swimming (4.7%). In addition, aerobic activities like step, zumba, and pilates, as well as community-based outdoor activities, were each practiced by 3.6% of students. Finally, cycling represented the least common form of activity, reported by 2.7% of the participants. Among the participants, 8.4% reported other type of recreational activities. These findings suggest that university students tend to prefer team-based and accessible individual physical activities, such as ball sports, running, and fitness training, while participation in more structured or specialized activities such as combat or water sports remains relatively low.

Participation patterns further showed that recreational activities were primarily peer-oriented: 63.3% of students preferred engaging in activities with friends, whereas 23.3% participated individually and only a small minority reported participating with their families. Residential status aligned with this trend, as most students lived in dormitories (45.7%) or with their families (39.3%), while fewer lived alone (7.5%) or with friends (7.5%). Notably, despite many students residing with their families, very few engaged in recreational activities with them. This suggests that university students construct their leisure experiences largely within peer-based social environments, emphasizing the central role of friendships and group participation in shaping their recreational behaviors.

In Table 1, as presented all variables have skewness and kurtosis values within the acceptable ±3 range, indicating that the data distributions are approximately normal (Kline, 2011; George and Mallery, 2019). RFES (Recreational Flow Experience Scale) shows a slightly negative skewness and higher kurtosis, suggesting a slightly peaked shape. SCS (Social Connectedness Scale) has a mild positive skewness, while BRS (Psychological Resilience Scale) and LSS (Life Satisfaction Scale) are nearly symmetric. All scales demonstrated acceptable levels of internal consistency (see Table 1). These coefficients indicate that all instruments demonstrated acceptable to excellent reliability within the sample. Therefore, the dataset met the assumptions required for subsequent parametric analyses, including mediation testing using Hayes’ PROCESS macro. Preliminary data screening indicated no substantial missing data or influential outliers. Skewness and kurtosis values for all study variables were within acceptable limits, supporting the assumption of normal distribution. All VIF values were below the recommended threshold of 3.3, indicating that common method variance was not a serious concern.

**Figure 1:**
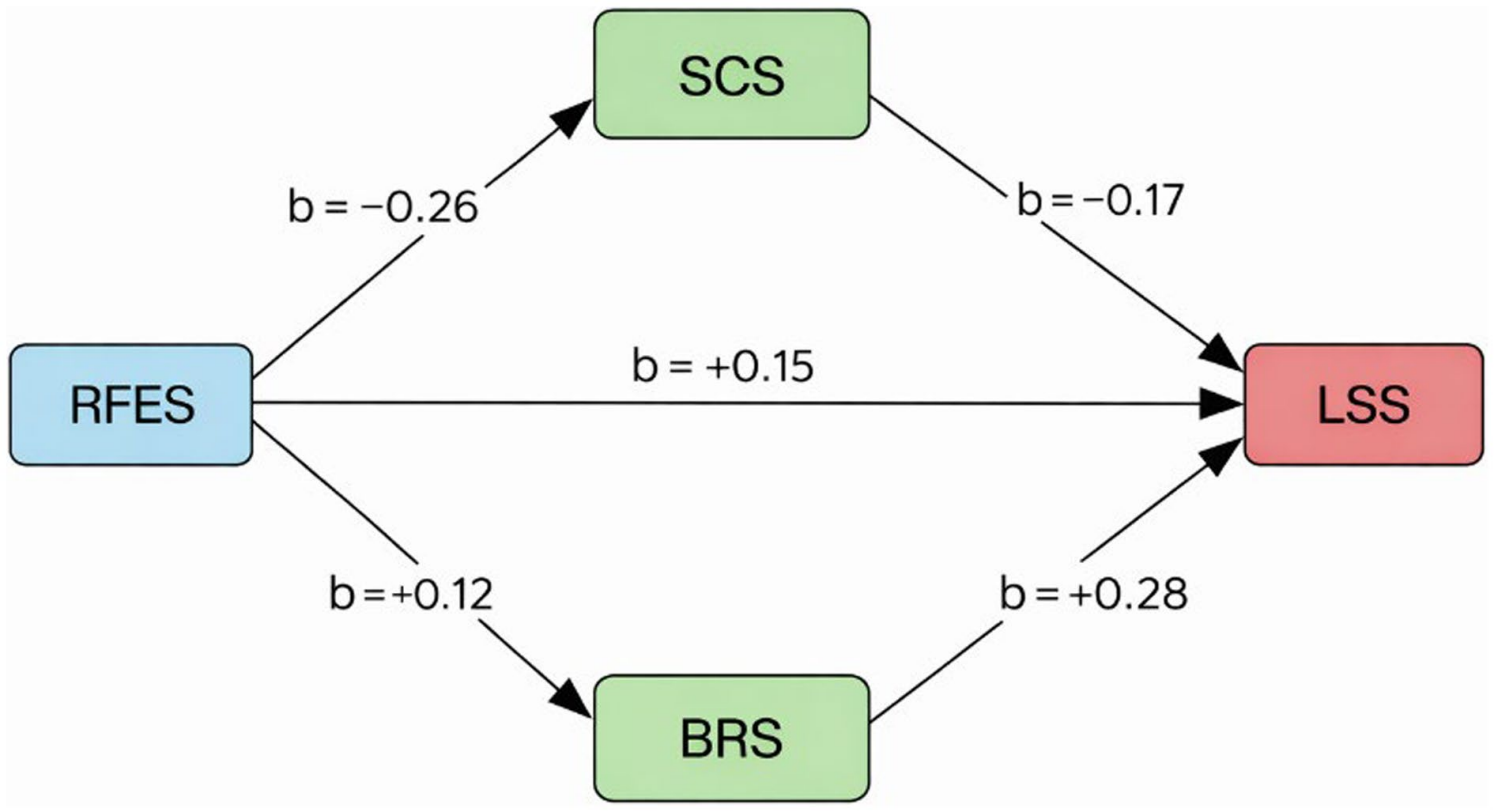
Path diagram of the mediation model with regression coefficients. The negative coefficients for social connectedness reflect the reverse-coded structure of the scale rather than an actual negative conceptual relationship. In the Turkish version (Duru, 2007), higher scores indicate stronger connectedness; therefore, these coefficients should be interpreted as positive associations in conceptual terms.

Figure 1 illustrates the direct and indirect relationships between recreational flow experience (RFES) and life satisfaction (LSS) through two mediators: social connectedness (SCS) and psychological resilience (BRS). The path from RFES to SCS (b = −0.26***) was statistically significant, representing a positive conceptual association between flow and social connectedness after accounting for reverse coding. The path RFES → BRS (b = +0.12***) indicated that greater flow experiences were related to higher psychological resilience. The path SCS → LSS (b = −0.17***) likewise reflected a conceptual positive association, while BRS → LSS (b = +0.28***) demonstrated the strongest direct effect on life satisfaction. Finally, the direct path RFES → LSS (b = +0.15***) confirmed that recreational flow exerts an independent positive influence on life satisfaction.

Table 2 presents the direct path coefficients between recreational flow experience (RFES), social connectedness (SCS), psychological resilience (BRS), and life satisfaction (LSS). The path from RFES to SCS was statistically significant (b = −0.26, *p* < 0.001). Recreational flow significantly predicted social connectedness (b = −0.26, *p* < 0.001). The path RFES → BRS (b = 0.12, *p* < 0.001) was positive and significant, suggesting that greater flow experiences enhance psychological resilience. The direct effect of RFES on LSS (b = 0.15, *p* < 0.001) confirmed that recreational flow independently contributes to higher life satisfaction.

**Table 2:** Direct effects between RFES, SCS, BRS, and LSS.

Dependent variable	Predictor	b	SE	t	*p*	LLCI	ULCI
SCS (M₁)	RFES	−0.2648	0.0328	−8.06	**<0.001**	−0.329	−0.200
BRS (M₂)	RFES	0.1193	0.0170	7.01	**<0.001**	0.086	0.153
LSS (Y)	RFES	0.1466	0.0224	6.54	**<0.001**	0.103	0.191
	SCS	−0.1660	0.0242	−6.87	**<0.001**	−0.214	−0.119
	BRS	0.2824	0.0466	6.06	**<0.001**	0.191	0.374

Bold values indicate statistically significant results (*p*< 0.001).

Regarding the mediators, SCS showed a negative coefficient (b = −0.17, *p* < 0.001) due to reverse coding; conceptually, however, this reflects that higher social connectedness is associated with greater life satisfaction. BRS exhibited a strong positive effect (b = 0.28, *p* < 0.001), indicating that resilience serves as a more robust predictor of life satisfaction compared to connectedness.

Table 3 presents the indirect effects of recreational flow experience (RFES) on life satisfaction (LSS) tested through Hayes PROCESS Model 4 using 5,000 bootstrap samples. The results show that the total indirect effect of RFES on LSS is significant (b = 0.0777, 95% CI [0.0536, 0.1053]). Specifically: The indirect effect through social-connectedness (SCS) is significant (b = 0.0440, 95% CI [0.0258, 0.0651]). The indirect effect through psychological resilience (BRS) is also significant (b = 0.0337, 95% CI [0.0201, 0.0501]). Since all confidence intervals exclude zero, both mediators have statistically significant indirect effects.

**Table 3:** Indirect effects of RFES on LSS (mediation analysis results, bootstrap = 5,000).

Intermediary variable	Indirect effect (b)	Boot SE	LLCI	ULCI
Total indirect effect	0.0777	0.0132	0.0536	0.1053
SCS	0.0440	0.0101	0.0258	0.0651
BRS	0.0337	0.0077	0.0201	0.0501

Table 4 presents the predictive effects of recreational flow experience (RFES), social-connectedness (SCS), and psychological resilience (BRS) on life satisfaction (LSS) based on the Hayes PROCESS Model 4 results. Findings indicate that: RFES → LSS shows a positive and significant effect (b = 0.15, *p* < 0.001), implying that higher flow experiences are associated with greater life satisfaction. SCS → LSS exhibits significant relationship (b = −0.17, *p* < 0.001). Although the coefficient appears negative, this direction arises from the reverse-coded wording of the SCS items (Duru, 2007). Conceptually, this result reflects a positive association between social connectedness and life satisfaction—students with stronger social ties and a greater sense of belonging reported higher satisfaction with life. BRS → LSS demonstrates a strong positive effect (b = 0.28, *p* < 0.001), indicating that resilience is the strongest predictor of life satisfaction among the tested variables. The model was found to be significant (R^2^ = 0.226; *F* (3,806) = 78.36, *p* < 0.001).

**Table 4:** Predictive effects of RFES, SCS, and BRS on life satisfaction (LSS) (Hayes PROCESS model 4 results).

Predictor	B	SE	t	*p*	LLCI	ULCI	R^2^
RFES	0.1466	0.0224	6.54	<0.001	0.103	0.191	0.226
SCS	−0.1660	0.0242	−6.87	<0.001	−0.214	−0.119	
BRS	0.2824	0.0466	6.06	<0.001	0.191	0.374	

## 5 Discussion

The findings of this study provide empirical support for the hypothesized relationships between recreational flow experience, social connectedness, psychological resilience, and life satisfaction among university students. All five hypotheses (H1–H5) were supported (Table 5), indicating that recreational flow is associated with life satisfaction both directly and indirectly through social and psychological pathways.

**Table 5:** Summary of hypothesis testing results.

Hypothesis	Path/relationship	Figure/table reference	Result
H1	recreational flow → social connectedness	Figure 1; Table 2	Accepted (b = −0.26, *p* < 0.001) *reverse-coded; conceptually positive*
H2	recreational flow → psychological resilience	Figure 1; Table 2	Accepted (b = 0.12, *p* < 0.001)
H3	social connectedness → life satisfaction	Figure 1; Tables 2,4	Accepted (b = −0.17, *p* < 0.001) *reverse-coded; conceptually positive*
H4	psychological resilience → life satisfaction	Figure 1; Tables 2,4	Accepted (b = 0.28, *p* < 0.001)
H5	recreational flow → (SCS & BRS) → life satisfaction (Parallel Mediation)	Figure 1; Table 3	Accepted (SCS b = 0.0440; BRS b = 0.0337; Total b = 0.0777)

Table 5 provides an integrated summary of the direct and indirect effects to facilitate interpretation of the overall mediation structure.

The first hypothesis, proposing a positive association between recreational flow and perceived social connectedness, was supported. This finding is consistent with previous research suggesting that engaging leisure participation is associated with higher levels of social interaction and involvement. Importantly, the present results do not imply that flow itself operates as a collective or social psychological state. Rather, they indicate that individual experiences of deep absorption and engagement during recreational activities are associated with higher perceived social connectedness among university students. Prior studies have shown that participation in shared recreational activities has been associated with greater opportunities for interaction and feelings of belonging (Duman et al., 2022; Ekinci and Gürbüz, 2021). In these contexts, flow is conceptualized as an individual experiential state that may occur during recreational participation, which often takes place in socially interactive settings where opportunities for interpersonal interaction are present. Accordingly, the observed association should be interpreted as reflecting a convergence between individual experiential engagement (as captured by the recreational flow measure) and perceived inclusion and belonging (as captured by the social connectedness scale), rather than as evidence that flow itself becomes a social or collective construct.

Thus, the present findings indicate that recreational flow is statistically associated with perceived social connectedness among university students. However, this association should not be interpreted as evidence that the psychological state of flow itself produces social connectedness. Rather, it may reflect the socially interactive nature of recreational participation, in which students frequently engage in leisure activities alongside peers and share recreational experiences. Previous research similarly suggests that campus recreation and informal leisure participation are associated with peer interaction and students’ sense of belonging within university environments (Eubank and DeVita, 2024; Freeman et al., 2024; Sim et al., 2026).

The second hypothesis, proposing a positive association between recreational flow and psychological resilience, was supported. This finding is consistent with prior research indicating that immersive and engaging leisure experiences are linked to adaptive coping and emotional regulation, which are central components of psychological resilience. Importantly, the present results should be interpreted with conceptual caution regarding the nature of flow experiences assessed in everyday recreational contexts. Classical flow theory conceptualizes flow as a relatively rare and intense optimal experience (Csikszentmihalyi, 1974). In contrast, everyday leisure activities are more likely to evoke flow-like or experiences, characterized by sustained engagement, enjoyment, and focused attention. Accordingly, the recreational flow experience measured in this study is best understood as reflecting frequent experiential engagement during leisure, rather than peak or extraordinary flow states. This distinction is important for interpreting the observed association with resilience.

From an empirical perspective, previous studies have shown that engagement in absorbing and rewarding recreational activities is associated with greater emotional regulation, perceived wellness, and adaptive functioning (Demiral and Karakaş, 2024; Özsoy et al., 2025; Dilmaç and Tezcan, 2024; Sarol et al., 2024). These findings suggest that regular exposure to engaging leisure experiences may coincide with stronger resilience-related capacities. However, consistent with Csikszentmihalyi’s (1974) original formulation, the present study does not assume or demonstrate a clear causal direction between flow-related experiences and positive psychological states. It remains equally plausible that individuals with greater adaptive capacity or more positive affective dispositions are more likely to experience flow-like engagement during recreation.

Rather than positioning recreational flow as a causal generator of resilience, the current findings indicate a robust association between experiential engagement in recreation and psychological resilience. From this perspective, flow-oriented recreational involvement can be understood as part of a broader psychosocial pattern in which engaging experiences, adaptive regulation, and coping capacity co-occur. This interpretation aligns with existing literature emphasizing that flow-related characteristics (e.g., deep involvement and reduced self-consciousness) are descriptively associated with adaptive functioning, without requiring strict assumptions about causal precedence. Overall, the results suggest that recreational engagement characterized by sustained experiential absorption is meaningfully linked to resilience among university students, while acknowledging the conceptual and empirical challenges inherent in disentangling causes and effects in flow research.

The third hypothesis was supported, indicating that higher levels of social connectedness were associated with greater life satisfaction among university students. This finding is consistent with prior empirical evidence showing that perceived social connectedness explains variance in life satisfaction beyond demographic and background factors in undergraduate populations (Blau et al., 2016). Importantly, the present results extend this evidence by situating social connectedness within a recreational flow framework, suggesting that feelings of belonging and inclusion fostered through leisure contexts are meaningfully linked to students’ global life evaluations. In this sense, the current findings align with and reinforce Blau et al.’s conclusion that social connectedness represents a central psychosocial resource for life satisfaction during the university years.

The fourth hypothesis, proposing a positive association between psychological resilience and life satisfaction, was supported. This finding indicates that university students who reported higher levels of resilience also tended to evaluate their lives more positively. Within the present model, psychological resilience emerged as a salient intrapersonal resource associated with favorable cognitive evaluations of life, consistent with subjective well-being research emphasizing adaptive functioning under stress. Previous empirical studies have consistently demonstrated that resilience is linked to life satisfaction by supporting emotional stability, adaptive coping, and psychological balance, particularly in populations exposed to academic and developmental stressors. For example, research conducted with university students has shown that individuals with higher resilience report greater life satisfaction and lower psychological distress, highlighting resilience as a key factor in sustaining positive life evaluations (Kapıkıran and Acun-Kapıkıran, 2016; Al-Abyadh and Azim, 2020).

Importantly, resilience is not conceptualized here as a mechanistic explanatory model, but rather as a capacity that enables individuals to manage challenges, recover from setbacks, and maintain psychological equilibrium. Empirical evidence suggests that such capacities are strengthened through active engagement with challenging life experiences that foster emotional regulation, coping competence, and psychological flexibility (Qiu et al., 2025). These adaptive processes are particularly relevant in university contexts, where students must navigate ongoing academic demands and social transitions.

The fifth set of hypotheses, which proposed that social connectedness and psychological resilience would operate as parallel mediators in the association between recreational flow and life satisfaction, was supported. By demonstrating a significant association between psychological resilience and life satisfaction while accounting for recreational flow and social connectedness, the present findings extend prior research by situating resilience within a broader psychosocial system. This perspective underscores resilience as a foundational psychological resource that supports positive life evaluations among university students, operating alongside experiential engagement and social integration rather than as an isolated determinant. The findings supported the proposed parallel mediation model, indicating that social connectedness and psychological resilience jointly transmit the association between recreational flow and life satisfaction. This result suggests that recreational flow is linked to life satisfaction not only through direct experiential engagement, but also through complementary interpersonal and intrapersonal psychosocial pathways.

Consistent with prior research, flow experiences have been associated with well-being and with psychosocial resources rather than merely with transient positive states (Bayram et al., 2025; Song et al., 2024). In the present model, social connectedness represents an interpersonal factor that is statistically associated with recreational flow and life satisfaction, whereas psychological resilience reflects an intrapersonal factor related to adaptive coping and emotional regulation. Importantly, these mediators operated in parallel, underscoring that flow-oriented recreation may simultaneously support social integration and psychological strength in university students.

Together, these findings extend existing leisure and well-being research by empirically demonstrating that recreational flow is associated with life satisfaction through multiple, complementary psychosocial pathways rather than through a single explanatory pathway.

### 5.1 Overall discussion and synthesis

Taken together, the findings of the present study indicate that recreational flow experiences are meaningfully associated with university students’ life satisfaction through both interpersonal and intrapersonal psychosocial resources. By integrating experiential engagement (recreational flow) with social connectedness and psychological resilience within a single parallel mediation model, the study highlights recreational flow experience as part of a broader psychosocial system supporting well-being during the university years.

Across hypotheses, the results consistently suggest that recreational flow is not merely a transient experiential state, but one that co-occurs with stronger perceptions of social integration and greater adaptive psychological capacity. In this sense, the model demonstrates that life satisfaction is shaped by multiple, complementary pathways: an interpersonal pathway reflecting perceived belonging and inclusion, and an intrapersonal pathway reflecting adaptive coping and resilience. The parallel operation of these pathways underscores the multidimensional nature of well-being in higher education contexts.

Importantly, the present findings also showed that recreational flow retained a significant direct association with life satisfaction even when social connectedness and psychological resilience were simultaneously included as mediators. This result is consistent with prior research conducted among undergraduate populations, which has shown that leisure-related flow experiences are positively associated with life satisfaction across multiple life domains (Hou and Jiang, 2023). The current study extends this evidence by demonstrating that this direct association remains robust within a more comprehensive psychosocial framework, suggesting that experiential engagement in recreation contributes to students’ global life evaluations both independently and through associated social and psychological resources.

### 5.2 Contextual observations on recreational participation

Although recreational activity types were not analyzed as predictors within the structural model, descriptive findings indicated that students predominantly engaged in socially oriented forms of recreation and that a majority preferred participating in leisure activities with friends. These patterns are presented as contextual background rather than inferential evidence. Nevertheless, they provide a meaningful backdrop for interpreting the central role of social connectedness observed in the model, by illustrating that university students’ recreational experiences are frequently embedded in peer-based and socially interactive contexts.

### 5.3 Theoretical integration

From a theoretical standpoint, the findings align with core assumptions of positive psychology (Seligman, 2011) and the subjective well-being perspective (Diener, 1984), which emphasize engagement, adaptive psychological functioning, and meaningful social relationships as central components of a satisfying life. In this context, the constructs examined in the present study correspond closely to key elements of these theoretical perspectives. Recreational flow reflects experiential engagement, social connectedness represents relational well-being, and psychological resilience captures adaptive psychological strength. Together, these resources contribute to students’ global evaluations of life satisfaction, consistent with the multidimensional understanding of well-being proposed in positive psychology and subjective well-being frameworks.

However, rather than positioning these frameworks as mechanistic explanatory models, the present study uses them as conceptual lenses to interpret how experiential, interpersonal, and intrapersonal resources co-occur in relation to life satisfaction. In this respect, recreational flow is best understood not as a singular causal driver, but as an experiential context in which social integration and psychological resilience may co-occur alongside favorable life evaluations.

Overall, the findings indicate that flow experiences are associated with life satisfaction alongside complementary interpersonal and intrapersonal psychosocial factors. Rather than positioning recreation itself as a causal agent, the results highlight how experiential engagement characterized by flow is associated with perceived social connectedness and psychological resilience among university students.

## 6 Conclusion

This study examined the direct and indirect associations between recreational flow experience and life satisfaction among university students, with social connectedness and psychological resilience tested as parallel mediators. The findings supported all hypotheses: higher recreational flow was linked to greater social connectedness and psychological resilience, which were each related to higher life satisfaction. Together, these results indicate that recreational flow is connected to life satisfaction through complementary interpersonal (social connectedness) and intrapersonal (psychological resilience) pathways in the university student population.

From a theoretical standpoint, these results support the core assumptions of positive psychology and the subjective well-being framework, which emphasize that flourishing arises from engagement, positive relationships, and the development of adaptive strengths. The present study extends these frameworks by identifying recreational flow as a meaningful experiential context in which psychosocial resources, such as social belonging and resilience, may co-occur alongside higher life satisfaction.

### 6.1 Practical implications

The findings have actionable implications for university student affairs and campus recreation units seeking to enhance student well-being. Because recreational flow was associated with life satisfaction through both social connectedness and psychological resilience, universities may consider recreation not only as leisure provision but also as a context that can support psychosocial resources.

First, campus recreation programming may be designed to increase the likelihood of flow by addressing factors beyond the challenge–skill balance.

Second, to support opportunities for social connectedness within recreational settings, universities can intentionally build “connection architecture” into recreation: small stable groups, cooperative tasks, peer-mentoring formats, and inclusive onboarding practices for newcomers. Importantly, these practices do not assume that flow itself is a social process; rather, they recognize that individual flow experiences often occur within shared recreational settings where interaction, cooperation, and repeated participation may support the development of social connectedness. Activities such as cooperative sports, creative arts groups, music ensembles, outdoor and nature-based group recreation, and challenge-based team events can be implemented with explicit belonging goals (e.g., rotating partners, shared group goals, and brief post-activity reflection). Third, to leverage resilience benefits, programs can include manageable challenges and mastery experiences that allow students to experience progress and recovery after setbacks in a low-stakes environment (e.g., graded skill challenges, goal-setting with feedback, and debriefs that normalize effort and learning). Student life practitioners can also integrate brief psychoeducation elements (e.g., coping strategies, self-efficacy prompts) without turning recreation into therapy, maintaining the intrinsic nature of the activity.

Overall, these results suggest that campus recreation providers can apply a positive-psychology-informed approach by designing recreational settings that promote engaging recreational experiences, create opportunities for social interaction and belonging, and support adaptive coping - factors that may be associated with higher life satisfaction among university students.

### 6.2 Limitations and future research

Several limitations should be acknowledged. First, the cross-sectional design limits causal interpretation of the observed relationships. Second, the reliance on self-report measures may increase the risk of shared method bias. Third, although a parallel mediation model was tested, potential sequential relationships between social connectedness and psychological resilience were not examined.

Future research could address these limitations by adopting longitudinal and experimental approaches. For example, intervention studies comparing structured, flow-supportive recreation programs with standard recreational activities may help clarify causal pathways. Future experiments could also intentionally manipulate key antecedents of flow—such as goal clarity, autonomy in activity choice, or the level of environmental distraction—to examine how specific design features influence flow experiences during recreational activities. Longitudinal designs could further explore whether sustained engagement in flow-based recreation is associated with changes in social connectedness, psychological resilience, and life satisfaction over time.

In addition, future studies may examine other relevant mechanisms, such as mindfulness, self-efficacy, or the personal meaningfulness of leisure activities, to better understand when and for whom recreational flow contributes most strongly to well-being.

## Data Availability

The datasets presented in this article are not readily available because due to confidentiality agreements and ethical restrictions. Requests to access the datasets should be directed to sevim.gullu@iuc.edu.tr.
